# AMBRA1, Autophagy, and the Extreme Male Brain Theory of Autism

**DOI:** 10.1155/2019/1968580

**Published:** 2019-10-10

**Authors:** Bernard Crespi, Silven Read, Amy Ly, Peter Hurd

**Affiliations:** ^1^Department of Biological Sciences, Simon Fraser University, Burnaby, British Columbia, Canada; ^2^Department of Psychology and Centre for Neuroscience, University of Alberta, Edmonton, Canada

## Abstract

The extreme male brain theory of autism posits that its male bias is mediated by exaggeration of male-biased sex differences in the expression of autism-associated traits found in typical populations. The theory is supported by extensive phenotypic evidence, but no genes have yet been described with properties that fit its predictions. The autophagy-associated gene AMBRA1 represents one of the top genome-wide “hits” in recent GWAS studies of schizophrenia, shows sex-differential expression, and has been linked with autism risk and traits in humans and mice, especially or exclusively among females. We genotyped the AMBRA1 autism-risk SNP in a population of typical humans who were scored for the dimensional expression of autistic and schizotypal traits. Females, but not males, homozygous for the GG genotype showed a significant increase in score for the single trait, the Autism Quotient-Imagination subscale, that exhibits a strong, significant male bias in typical populations. As such, females with this genotype resembled males for this highly sexually dimorphic, autism-associated phenotype. These findings support the extreme male brain hypothesis and indicate that sex-specific genetic effects can mediate aspects of risk for autism.

## 1. Introduction

Autism exhibits a strong male bias in its incidence, that increases from about 2 : 1 in relatively severe, monogenic cases to about 5–10 : 1 among autistic individuals with relatively high intelligence [1, 2]. The expression of autism phenotypes also shows evidence of differences between females and males, although the patterns of sex differences remain unclear. Sex differences in autism are important because they provide information regarding its potential causes, which either differ by sex or exert effects that depend upon the degree to which males exhibit increased vulnerability and females show increased protection, for autistic traits and diagnoses [3].

Asperger [4] suggested that autism represents “an extreme form of male intelligence” and “character.” Baron-Cohen [5] developed this idea into the “extreme male brain” hypothesis of autism, which posits that autism expression and risk are associated with brain “masculinization” due to high levels of prenatal testosterone. High prenatal testosterone in turn drives altered expression of a suite of sex-differential phenotypes, especially those related to empathizing (empathy towards people, which is female-based in typical individuals) and systemizing (interest in mechanistic, rule-based systems, which is male-based in typical individuals). By the extreme male brain hypothesis, autism is thus typified by low empathizing and high systemizing, in both females and males, as recently confirmed in large samples [6].

A recent genome-wide study of empathizing and systemizing reported that autism was, as predicted, negatively genetically correlated with Empathy Quotient scores and positively genetically correlated with Systemizing Quotient scores [7]. Moreover, the genes that contribute to high systemizing in males also tend to show male-biased expression in the brain [7]. These results support aspects of the extreme male brain theory in a genome-wide context, although the links of specific genes with sex differences and autism risk remain to be discerned.

Evaluation of a role for human sexual dimorphism in risk of autism can also involve tests to determine if specific autism-associated genes mediate shifts in the male “direction,” for psychological or other traits that show sex differences in typical populations. Such effects may manifest in males, females, or both sexes and thus also provide information regarding sex-specificity of genetic effects that generate insights into sex differences in the expression of different autism phenotypes.

The gene AMBRA1 (Activating Molecule in Beclin 1-Regulated Autophagy) codes for a gene product that shows sex-differential expression and has been linked to autism-related and schizophrenia-related phenotypes, exclusively or predominantly among females, in both humans and mice [8–11]. AMBRA1, moreover, represents one of the top genome-wide significant genes for risk of schizophrenia in recent GWAS studies [12, 13]; as such, determining the role of this gene, in autism- and schizophrenia-associated psychological and neurological phenotypes, is crucial to evaluating its functions.

Recently, Mitjans et al. [10] showed that the AMBRA1 SNP rs3802890 was associated with measures of sociality in a cohort of individuals with schizophrenia, and in a cohort of typical individuals, with significant effects limited to females in both populations. Different SNPs of AMBRA1 have also been linked with risk of schizophrenia, although sex differences were not investigated here [13]. The AMBRA1 gene is of further interest in the context of psychological sex differences because it mediates the cellular process of autophagy, cellular recycling. Autophagy modulates neurodevelopment, with striking sex differences in its neuronal effects [11].

In this study, we genotyped the AMBRA1 SNP rs3802890 in a population of typical individuals, to test the hypothesis that this SNP affects autism-related traits in a female-specific manner (and potentially schizophrenia-related traits as well), as found in previous studies and subject to replication and extension here. This study also serves to characterize the psychological effects from an important genomic-wide significant gene that is implicated in neurodevelopment.

## 2. Methods

This research was approved by the ethics boards of the University of Alberta (Pro00015728) and Simon Fraser University (2010s0554), and all participants provided written informed consent. We collected the questionnaire and DNA data from 531 undergraduate students (308 females and 223 males). Levels and forms of schizotypal traits were quantified using the Schizotypal Personality Questionnaire-Brief Revised [14]. This questionnaire comprises 32 items using a 5-point Likert scale, with response choices that range from “strongly disagree” to “strongly agree.” It includes seven subscales: (1) constricted affect, (2) social anxiety, (3) magical thinking, (4) unusual perceptions, (5) ideas of reference, (6) eccentric behavior, and (7) odd speech, which sum to total schizotypy. The Autism Spectrum Quotient (AQ) [15] was used to quantify the extent to which participants endorsed autism spectrum phenotypes. This is a 50-item questionnaire that assesses autistic traits across five domains including (1) sociality, (2) communication, (3) attention to detail, (4) attention switching, and (5) imagination, with the total AQ score as the sum.

DNA was extracted from saliva using standard phenol-chloroform protocols. Fluorophore-labelled primers for rs3802890 (position in human genome version GRCh38.12, Chr11 : 46512996) were used in TaqMan genotyping using a Roche LightCycler 96 Real-Time PCR machine. Fluorescence data were analyzed under Endpoint Genotyping using LightCycler 96 software, v. 1.1.0.1320, and genotyping success was over 97%.

Genotypes were in the Hardy–Weinberg equilibrium (*χ*^2^ = 0.003, *p* > 0.50), and minor allele frequency (allele G) in the population was 0.30, closely similar to that of the 1000 Genomes European Superpopulation (0.31). Statistics were conducted in R v. 3.5.3 [16]. Sexes were analyzed separately based on strong *a priori* expectations from previous studies that reported sex-differential effects, as described above. The false discovery rate (FDR) was used to adjust for multiple testing. Post hoc tests were calculated using the pooled variance pairwise.*t*.test() function. All tests were 2-tailed.

## 3. Results

### 3.1. Sex Differences in AQ and SPQ Scores

The autism subscale AQ-Imagination showed a significant male bias (*p*=0.0016 unadjusted and 0.0095 after FDR adjustment), and the schizotypy subscales SPQ-Magical Thinking, SPQ-Ideas of Reference, and SPQ-Odd Speech each showed significant female biases after FDR adjustment (Table 1). None of the other AQ or SPQ subscales showed significant sex biases after FDR adjustment.

### 3.2. Genotype Differences in AQ and SPQ Scores

For the AQ, females showed nominally significant differences between the three genotypes for the AQ-Social (*p*=0.014), AQ-Communication (*p*=0.041), and the AQ-Imagination (*p*=0.0009) subscales (Table 1). Only the AQ-Imagination difference remained significant (*p*=0.022) after FDR adjustment. There were no differences for any of the AQ subscales among males (lowest *p* value: 0.738).

Post hoc tests suggested that the difference among genotypes for females, for the AQ-Imagination subscale, was attributable predominantly to high scores on this subscale for GG females (Table 1; Supplementary [Supplementary-material supplementary-material-1]). Females with the GG genotype showed average scores on the AQ-Imagination subscale (3.04) that were higher than those of GG males (2.81), though nonsignificantly so (*t*-test, *P* > 0.5). By contrast, AA + AG males showed AQ-Imagination scores that were highly significantly greater (average: 2.51) than those of AA + AG females (average: 2.00; *P*=0.00056 unadjusted and 0.0067 after FDR adjustment). These results demonstrate that GG females exhibit male-typical scores on the AQ-Imagination subscale. Females with the GG genotype did not differ significantly in AQ-Imagination scores when compared to the entire male population including all three genotypes (*t* = 1.47, *p*=0.14).

For the SPQ, neither sex showed genotypic differences, for any subscale (lowest *p* values: 0.7 for females and 0.17 for males) (Table 1). There were no significant, FDR-adjusted genotypic differences, for either the SPQ or the AQ subscales, for analyses with the sexes combined.

## 4. Discussion

The main findings of this study are twofold. First, the AMBRA1 SNP rs3802890 genotype showed strong sex specificity in its phenotypic effects, with restriction to females. These results corroborate and extend previous analyses of mice and humans [8, 10, 17], all of which show female-restricted or female-biased effects on psychological, psychiatric, and neurological phenotypes.

Second, the sex-limited genotypic effects observed here are confined to a single AQ subscale, AQ-Imagination, for which GG females exhibited higher (more autistic) scores than did AA combined with AG females. Two other subscales, AQ-Social and AQ-Communication, showed nominally significant genotypic differences; this finding is of interest because these three subscales, AQ-Imagination, AQ-Social, and AQ-Communication, represent the metrics that best reflect social skills and interests, whereas the other two AQ subscales center on aspects of attention. As such, these results are consistent with those of the previous work on AMBRA1 individuals scored for rs3802890, where social phenotypes were affected [10].

With respect to predictions from the extreme male brain hypothesis, the main finding reported here is that individuals with the GG genotype showed a lack of expected male bias for AQ-Imagination because the scores of GG females were similar to those of GG males for this, the only sexually dimorphic, male-biased phenotype in the AQ. Indeed, AA + AG males showed substantially and significantly higher scores than AA + AG females on the AQ-Imagination subscale.

Restriction of strong, significant genotypic effects to AQ-Imagination is intriguing because this subscale shows the strongest male bias of all of the AQ subscales, in the typical population studied here. This same pattern was reported in a meta-analysis of 11 populations, which showed that the male bias in the AQ-Imagination subscale is about twofold higher than the male bias for AQ-Social and AQ-Communication and still higher than that for AQ-Switching and AQ-Detail [18]. The questions in the AQ-Imagination subscale have been described as reflecting functions of the brain's default mode, which subserves social narrative processing, social play, social mental imagery, and mentalizing more generally [18]. The default mode network also shows replicated sex differences in connectivity [19–21], with typical males lower in connectivity than typical females, and individuals with autism lower than typical individuals, with the sex differences mediated in part by fetal testosterone [20]. Imaging genetics would be useful to test the hypothesis that the AMBRA1 SNP rs3802890 genotype modulates human default mode phenotypes, selectively in females.

What molecular and developmental mechanisms might mediate the effects of AMBRA1 genotype described here? AMBRA1 regulates autophagy, which plays central roles in neurodevelopment through effects on several processes [11, 22, 23], including mTOR activation [9, 24, 25] and synaptic pruning via microglial activity [26, 27]. Loss of function in multiple genes that negatively regulate mTOR, including TSC1, TSC2, PTEN, NF1, and FMR1, has been linked to syndromic autism with increased head size and high rates of seizures [28]; both of these traits are also found in mice heterozygous for AMBRA1 loss of function [17]. Idiopathic autism and syndromic autism commonly involve increased mTOR activity [29, 30].

Reduced synaptic pruning and increased dendritic spine density have been described as strong correlates of autism [31, 32]; in mice, heterozygotes for loss of AMBRA1 function show female-restricted increases in dendritic spine density [17]. Tang et al. [33] demonstrated that reductions in mTOR-dependent autophagy lead to autistic-like synaptic pruning deficits in mice, and Zhang et al. [34] reported that valproic acid-induced autism rat models exhibit decreased autophagy and increased dendritic spine densities in conjunction with increased activity of the Notch pathway, which positively regulates mTOR [35–37]. These findings provide convergent evidence that high mTOR activity and concomitantly reduced autophagy are linked with reduced synaptic pruning, increased spine density, and the expression of autism phenotypes.

Human sex differences in autophagy are prominent in the brain and other tissues [38–41], with sex-differential effects from genetic variation also found in the autophagy-regulating gene ADNP (Activity-Dependent Neuroprotective Protein), which, like AMBRA1, affects autism and schizophrenia risk and traits [42–44]. Sex differences in autophagy are modulated in part by testosterone, and autophagic processing is especially active in the testosterone-secreting Leydig cells of the testis [45, 46]; testosterone levels are also increased in PTEN-deleted cells with overactivation of the mTOR pathway [47].

The SNP rs3802890 is linked only moderately with the SNPs identified by GWAS as associated with schizophrenia (with *r*^2^ values between 0.40 and 0.50), which may help to explain its lack of significant effects on schizotypal traits as found here. Given that schizophrenia has been characterized by reduced mTOR activation [48] and increased synaptic pruning [49], it might be expected to show a different pattern from that of autism with regard to AMBRA1 expression, activity, and direction of effects.

In conclusion, this study has provided support for the extreme male brain hypothesis of autism, in showing that females with the GG genotype for the SNP rs3802890, in the autophagy-activating gene AMBRA1, exhibit male-like, or male-exceeding, scores on AQ-Imagination, the most male-biased of subscales on the Autism Quotient test. These results are also important in that activation of autophagy has recently been shown to rescue cognitive and synaptic deficits in a mouse model of the autism-associated fragile X syndrome [50], suggesting that autophagy-related pathways have therapeutic potential.

## Figures and Tables

**Table 1:** Sex and genotype variation for the Autism Quotient and Schizotypal Personality Questionnaire subscales, for the AMBRA1 SNP rs3802890.

Questionnaires	Sex differences			Genotype differences by sex				
AQ and SPQ subscales	Sex	Mean (SD)	*t*, *p* (*p* after FDR adjustment)	Sex	Genotype AA, mean (SD) (Females *N* = 160, males *N* = 100)	Genotype AG, mean (SD) (Females *N* = 121, males *N* = 102)	Genotype GG, mean (SD) (Females *N* = 27, males *N* = 21)	*F*, *p* (*p* after FDR adjustment)
AQ-Social	Females Males	2.40 (2.25) 2.16 (2.09)	1.27, 020 (0.27)	Females Males	2.75 (2.45) 2.15 (2.27)	2.07 (1.95) 2.13 (1.97)	1.78 (1.93) 2.33 (1.85)	4.23, 0.014 (0.169) 0.08, 0.919 (0.978)

AQ-Communication	Females Males	2.34 (1.88) 2.07 (1.70)	1.75, 0.08 (0.15)	Females Males	2.57 (2.08) 2.12 (1.70)	2.01 (1.55) 2.00 (1.74)	2.44 (1.76) 2.14 (1.59)	3.23, 0.041 (0.326) 0.15, 0.863 (0.978)

AQ-Imagination	Females Males	**2.09 (1.58)** **2.54 (1.65)**	**3.18, 0.0016 (0.0095)**	Females Males	**2.14 (1.53)** 2.52 (1.68)	**1.81 (1.52)** 2.51 (1.63)	**3.04 (1.74)** 2.81 (1.57)	**7.16, 0.0009 (0.022)** 0.30, 0.738 (0.978)

AQ-Attention to Detail	Females Males	5.48 (2.17) 5.50 (1.99)	0.138, 0.89 (0.97)	Females Males	5.41 (2.26) 5.54 (1.94)	5.64 (2.03) 5.45 (1.98)	5.15 (2.23) 5.57 (2.31)	0.71, 0.494 (0.978) 0.06, 0.938 (0.978)

AQ-Attention Switching	Females Males	5.00 (2.03) 4.71 (1.81)	1.71, 0.088 (0.15)	Females Males	5.09 (2.14) 4.75 (1.77)	4.76 (1.91) 4.65 (1.85)	5.56 (1.85) 4.85 (1.90)	2.01, 0.136 (0.653) 0.15, 0.857 (0.978)

SPQ-Ideas of Reference	Females Males	17.06 (4.26) 16.16 (3.95)	**2.52, 0.012 (0.036)**	Females Males	17.15 (4.38) 15.93 (3.98)	16.60 (4.13) 16.28 (4.00)	18.63 (3.79) 16.67 (3.62)	2.59, 0.077 (0.460) 0.39, 0.677 (0.978)

SPQ-Constricted Affect	Females Males	14.75 (4.99) 15.39 (4.73)	1.51, 0.131 (0.20)	Females Males	14.72 (5.14) 15.29 (4.31)	14.69 (4.77) 15.40 (5.21)	15.15 (5.16) 15.81 (4.40)	0.10, 0.908 (0.978) 0.10, 0.901 (0.978)

SPQ-Eccentric Behavior	Females Males	11.96 (3.91) 11.96 (3.65)	0.009, 0.993 (0.99)	Females Males	12.16 (4.14) 11.99 (3.73)	11.73 (3.60) 12.03 (3.67)	11.19 (3.88) 11.52 (3.20)	1.21, 0.300 (0.978) 0.17, 0.843 (0.978)

SPQ-Social Anxiety	Females Males	11.81 (4.12) 11.17 (3.57)	1.99, 0.048 (0.11)	Females Males	11.98 (4.40) 10.98 (3.28)	11.52 (3.77) 11.21 (3.84)	12.33 (3.97) 11.81 (3.54)	0.62, 0.538 (0.978) 0.48, 0.616 (0.978)

SPQ-Magical Ideation	Females Males	**8.59 (3.76)** **6.98 (3.25)**	**5.26, 2.1 × 10 ** ^**−7**^ ** (2.5 × 10 ** ^**−6**^ ** )**	Females Males	8.73 (4.01) 6.88 (3.04)	8.28 (3.48) 7.01 (3.61)	9.15 (3.40) 7.33 (2.31)	0.81, 0.446 (0.978) 0.18, 0.840 (0.978)

SPQ-Perceptual Aberration	Females Males	10.29 (3.03) 10.15 (2.56)	0.59, 0.56 (0.66)	Females Males	10.42 (3.17) 10.15 (2.84)	10.05 (2.86) 10.18 (2.32)	10.63 (2.94) 10.05 (2.42)	0.71, 0.494 (0.978) 0.02, 0.978 (0.978)

SPQ-Odd Speech	Females Males	13.41 (3.02) 12.66 (2.93)	**2.88, 0.0042 (0.017)**	Females Males	13.48 (3.15) 12.28 (3.07)	13.27 (2.87) 13.05 (2.83)	13.59 (2.92) 12.52 (2.60)	0.21, 0.810 (0.978) 1.77, 0.172 (0.688)

Boldface text indicates differences that remain significant after FDR adjustments. Total autism and schizotypy scores were not significant for either sex, nominally or after FDR.

## Data Availability

The data used to support the findings of this study are available from the corresponding author upon request.

## Supplementary Materials

Supplementary Materials Supplementary Table 1: post hoc analysis of Autism Quotient and Schizotypal Personality Questionnaire variation by AMBRA1 SNP rs3802890 genotype and sex. One-way ANOVA *F* and *p* values are presented, and all df are (2, 305), with *p* values for the corresponding pairwise genotype comparisons. FDR adjustments were made across all 36 tests. These results are the *p* values from the pairwise.*t*.test() function in R, which compares all the groups within an ANOVA using the pooled variance (including those not in the pair). Values significant after FDR are in boldface.
