# Supplementary material for: AMBRA1, Autophagy, and the Extreme Male Brain Theory of Autism
Source: Autism Res Treat. 2019 Oct 10;2019:1968580. doi: 10.1155/2019/1968580 (PMC6811796; doi:10.1155/2019/1968580)
Supplement: Supplementary Materials — Supplementary Table 1: post hoc analysis of Autism Quotient and Schizotypal Personality Questionnaire variation by AMBRA1 SNP rs3802890 genotype and sex. One-way ANOVA F and p values are presented, and all df are (2, 305), with p values for the corresponding pairwise genotype comparisons. FDR adjustments were made across all 36 tests. These results are the p values from the pairwise.t.test() function in R, which compares all the groups within an ANOVA using the pooled variance (including those not in the pair). Values significant after FDR are in boldface. [file 1968580.f1.docx]

Supplementary Table 1. Post-hoc analysis of Autism Quotient and Schizotypal Personality Questionnaire variation by AMBRA1 SNP rs3802890 genotype and sex. One-way ANOVA F and p values are presented, and all df are (2, 305), with p-values for the corresponding pairwise genotype comparisons. FDR adjustments were made across all 36 tests. These results are the p-values from the pairwise.t.test() in R, which compares all the groups within an ANOVA using the pooled variance (including those not in the pair). Values significant after FDR are in boldface.

| AQ and SPQ Subscales | Sex | AA v AG  (p value, FDR) | AA v GG  (p value, FDR) | AG v GG  (p value, FDR) |
| --- | --- | --- | --- | --- |
| AQ-Social | Females  Males | 0.012 0.222  0.939 0.968 | 0.037 0.441  0.717 0.933 | 0.532 0.933  0.683 0.933 |
| AQ-Communication | Females  Males | 0.012 0.222  0.618 0.933 | 0.737 0.933  0.956 0.968 | 0.272 0.933  0.727 0.933 |
| AQ-Imagination | Females  Males | 0.074 0.590  0.965 0.968 | 0.0058 0.208  0.466 0.933 | **0.00023 0.016**  0.450 0.933 |
| AQ-Attention to Detail | Females  Males | 0.393 0.933  0.751 0.933 | 0.559 0.933  0.948 0.968 | 0.291 0.933  0.801 0.952 |
| AQ-Attention Switching | Females  Males | 0.181 0.933  0.688 0.933 | 0.268 0.933  0.806 0.952 | 0.066 0.590  0.630 0.933 |
| SPQ-Ideas of Reference | Females  Males | 0.285 0.933  0.525 0.933 | 0.094 0.679  0.439 0.933 | 0.025 0.366  0.687 0.933 |
| SPQ-Constricted Affect | Females  Males | 0.968 0.968  0.867 0.962 | 0.680 0.933  0.649 0.933 | 0.670 0.933  0.721 0.933 |
| SPQ-Eccentric Behavior | Females  Males | 0.264 0.933  0.939 0.968 | 0.186 0.933  0.596 0.933 | 0.509 0.933  0.565 0.933 |
| SPQ-Social Anxiety | Females  Males | 0.370 0.933  0.640 0.933 | 0.677 0.933  0.335 0.933 | 0.360 0.933  0.489 0.933 |
| SPQ-Magical Ideation | Females  Males | 0.328 0.933  0.777 0.949 | 0.589 0.933  0.563 0.933 | 0.280 0.933  0.679 0.933 |
| SPQ-Perceptual Aberration | Females  Males | 0.305 0.933  0.942 0.968 | 0.746 0.933  0.869 0.962 | 0.370 0.933  0.835 0.962 |
| SPQ-Odd Speech | Females  Males | 0.579 0.933  0.063 0.590 | 0.852 0.962  0.728 0.933 | 0.619 0.933  0.454 0.933 |
